# Risk factors for delayed healing and multiple revisions in humeral shaft nonunion: a retrospective case series

**DOI:** 10.1007/s00590-026-04785-z

**Published:** 2026-06-13

**Authors:** Ellen Lutnick, Timothy Whelan, Bradley Hawayek, Mikaela Hastings, Marco Flores, Kevin Schauer, Elias Joseph, Mohammad Nadir Haider, Matthew Binkley

**Affiliations:** 1 Department of Orthopaedic Surgery, University at Buffalo, State University of New York, Buffalo, USA; 2 Department of Orthopaedic Surgery, West Virginia State University, Dunbar, USA

**Keywords:** humeral shaft fracture, humeral fracture nonunion, nonunion, humeral shaft ORIF

## Abstract

**Purpose:**

To describe characteristics and outcomes of humeral shaft nonunion.

**Methods:**

Adult patients diagnosed with humeral shaft nonunion at one Level 1 trauma center from December 2000-July 2020 were retrospectively reviewed. Independent samples t-test was used for continuous variables, and chi squared or fishers exact test for categorical. *P* < 0.05 was considered significant.

**Results:**

59 patients (age 59.83 ± 17.71; 56.14 female; BMI 29.54 ± 8.5) had comorbidities included 32.70% current smoking, 14.30% alcohol use disorder, 21.40% hypothyroidism, 14.30% diabetes. 6 had open fractures; 42 (71.19%) were initially treated nonoperatively. 21 patients were diagnosed with atrophic nonunion, 4 with hypertrophic, and 28 with oligotrophic. 43 nonunions were treated surgically, 11 nonoperatively; the remainder were lost to follow up. 9 had positive cultures at revision. Those with negative revision cultures healed sooner (198 ± 177.54 vs. 451.25 ± 314.377 days, *p* = 0.034). 41 patients were treated with bone graft at revision. 42 patients (77.78%) required only one revision before radiographic healing; 12 required > 1. Patients with > 1 revision had longer follow-up (846.25 ± 624.75 vs. 358 ± 261.65 days, *p* < 0.001) and were slower to heal (433 ± 388.75 vs. 202.21 ± 157.38 days, *p* = 0.028); other factors were not significant on comparison.

**Conclusion:**

Humeral shaft nonunion remains a complex clinical problem. In this analysis, positive cultures at time of initial revision were found to significantly delay healing. Use of bone graft at time of revision was not significantly associated with faster healing or decreased likelihood of requiring > 1 revision procedure.

## Introduction

Tremendous variability has been described regarding humeral shaft nonunion rates, nonunion risk factors and treatments for nonunion. The reported rate of humeral shaft nonunion ranges from 2 to 33% for fractures initially treated nonoperatively and 15 to 30% for those treated operatively [1, 2].

Risk factors for nonunion are multifaceted, representing both systemic patient factors impacting healing as well as fracture-specific factors. Patient medical characteristics informing risk include obesity, alcohol excess, smoking, advanced age, and metabolic comorbidities [2–4]. Orthopaedic considerations include glenohumeral arthritis, osteoporosis, proximal diaphysis fracture location, transverse and short oblique fractures, open fractures, infections and preinjury NSAID use [1, 2, 5, 6].

Many authors define nonunion by a lack of signs of osseous healing on consecutive plain radiographs a minimum of six months after treatment start [5, 7, 8]. Some studies further describe radiographic signs of nonunion as the absence of fracture consolidation, increased interfragmentary diastasis or angulation and/or lack of bridging callus on at least 3 of 4 cortices [5, 8–10]. Other analyses diverge in terms of timing, diagnosing nonunion at a minimum of 12 weeks or between three and five months [9, 11, 12]. Clinically, nonunion is characterized by persistent pain and instability at the fracture site [7–9, 12].

Amongst nonunion, hypertrophic nonunion occurs in the setting of an adequate environment biologically and vascularly with insufficient mechanical stability and/or alignment [5, 13]. Conversely, atrophic nonunion results from inadequate vascular or biologic environment, recognized radiographically as the absence of callus [5, 13]. Oligotrophic nonunion lies between these entities, demonstrating minimal callus formation without resorption of the bone ends as observed in atrophic nonunion [14]. The current gold standard for treatment of humeral shaft nonunion is open reduction and internal fixation (ORIF) with compression plating and bone autograft, particularly for atrophic and oligotrophic nonunion [9]. Roddy et al. called into question the routine use of autograft, finding a union rate of 97% among those treated without autograft, insignificant compared to those treated with autograft [9]. Alternative treatments include intramedullary nailing with or without autogenous bone grafting, with described union rates of 88% and 66%, respectively, as reported by a systematic review by Peters et al. [8].

Taken together, the heterogeneity amongst available evidence illustrates the challenges associated with successful treatment of humeral shaft nonunion and reinforces the paucity of literature related to outcomes after nonunion treatment. This current study aims to characterize and evaluate the management approaches and subsequent recovery rates of a series of patients treated for humeral shaft nonunion.

## Methods

### Study design

This study is a single center, retrospective case series conducted at one academic Level 1 Trauma Center. The study was designed to evaluate the patient and treatment characteristics, including outcomes after treatment for humeral shaft nonunion in patients treated between December 1, 2000, and July 31, 2020.

### Inclusion and exclusion criteria

All adult patients (18 years and older) treated for humeral shaft nonunion were included, identified initially using the AO/OTA classification (12) and corresponding International Classification of Disease (ICD-10) codes within the specified time frame. AO/OTA classification was verified by orthopaedic surgery residents contributing to a Fracture Registry maintained by the institution. Patients were excluded if the proximal or distal extent of the fracture extended into the articular surface, if they underwent treatment at another facility, or had incomplete records regarding injury radiographs.

### Patient initial treatment

Patients initial treated nonoperatively were immobilized with coaptation splinting, followed by transition to Sarmiento bracing in an outpatient setting at approximately 1–2 weeks from the time of initial injury and splint placement. Occasionally, placement of a Sarmiento brace in the Emergency Department setting was performed per brace availability and various patient factors.

All patients who were initially treated with surgical intervention were temporized until the date of surgery with a plaster splint. Patients were treated via a variety of surgical techniques and approaches, including open reduction internal fixation with a plate and screws (ORIF), intramedullary nailing (IMN), or minimally invasive percutaneous osteosynthesis (MIPO).

### Data collection

Data were extracted from the electronic medical record using standardized chart review protocol, including demographic information, clinical and surgical characteristics, and radiographic analysis of the characteristics of nonunion. Nonunion was defined as either clinical evidence of persistent motion across the fracture site at 6 weeks or lack of evidence of bridging callus at 3 months on radiographic analysis [15]. Types of nonunion included hypertrophic (nonunion with radiographic callous without resorption of the bone ends of the fracture), atrophic (nonunion without callous formation including bony resorption at the fracture ends), and oligotrophic nonunion (nonunion with some callous formation, including bony resorption at the fracture ends). Specific variables included patient age, sex, body mass index (BMI), smoking status, comorbidities, mechanism of injury, and fracture classification according to the AO/OTA system. Treatment of nonunion included collection of data related to any revision or number of revision procedures, the use of bone grafting at time of revision, and data related to cultures taken at the time of revision.

### Outcome measures

The primary outcome was the treatment and outcome of treatment of diagnosed humeral shaft nonunion, including time to radiographic healing. Secondary outcomes included number of revision procedures and time to radiographic healing per treatment characteristics, including results from intraoperative cultures at the time of revision and use of bone graft at the time of revision. Radiographic analysis was performed by three senior orthopedic surgical residents, and any discrepancies were reviewed and decided upon by the senior author (MB).

### Statistical analysis

Patient characteristics and radiographic analyses were recorded. Descriptive statistics (mean and standard deviation for continuous variables, frequencies, and percentages for categorical variables) were calculated for the entire cohort and stratified by patient and treatment characteristics. Independent samples t-test was used for continuous variables, and chi squared or fishers exact test for categorical. *p* < 0.05 was considered significant. All analyses were performed using SPSS.

### Ethical considerations

The study was approved by the Institutional Review Boards (IRB) of the participating institution. All data collection and storage complied with HIPAA regulations, and all members of the research team were trained in data security and patient confidentiality.

## Results

Of 386 patients screened, a total of 59 patients met inclusion (age 59.83 ± 17.71; 56.14 female; BMI 29.54 ± 8.5) (Table 1). Comorbidities included current smoking status in 32.70%, 14.30% with documented alcohol use disorder, 21.40% hypothyroidism, 14.30% diabetes. 6 patients initially had open fractures; 42 (71.19%) were initially treated nonoperatively.

**Table 1:** Patient overview

		Mean	SD	Median	25th Percentile	75th Percentile	Count
P Age (Years)		59.83	17.71	63	48	71	
P Patient Sex	Male						27
	Female						32
BMI (kg/m^2^)		29.54	8.5	28.1	22.8	33.8	
Race	White						51
	African American						2
	Asian						2
	Hispanic						1
	American Indian						1
Hand dominance	Right						43
	Left						2
Smoking	Current						18
	Former						7
	Never						30
Occupation	Employed						15
	Unemployed						21
IDDM	Yes						3
	No						53
NIDDM	Yes						5
	No						51
Dialysis	Yes						0
	No						56
Alcohol use disorder	Yes						8
	No						48
Osteoporosis	Yes						1
	No						55
Hypothyroidism	Yes						12
	No						44
Mechanism	Low energy						38
	High energy						20
Side of injury	Left						32
	Right						27
Additional fracture							47
	Ipsilateral upper extremity						1
	Contralateral upper extremity						1
	Contralateral upper extremity + Lower extremity, pelvis						2
	Lower extremity, pelvis						3
	Lower extremity, pelvis + Spine						3
	Spine						2
Open fracture	Yes						6
	No						53
Associated injury	None						55
	Abdomen						1
	Chest						1
	Head						2
Treatment	Operative						17
	Non-operative						42

34 (57.63%) were mid-shaft humerus fractures. 15 were proximal third; 10 were distal third humeral shaft fractures (Table 2). 21 patients were diagnosed with atrophic nonunion, 4 with hypertrophic, and 28 with oligotrophic nonunion. 43 were treated surgically for their nonunion, 11 treated nonoperatively; the remainder of the cohort were lost to follow up.

**Table 2:** Nonunion characteristics, complications, and treatments

		Count
Fracture location	Proximal	15
	Mid-Shaft	34
	Distal	10
Intra-articular extension	No	58
	Yes	1
Complication	Radial nerve injury after injury	9
	Radial nerve injury after surgery	1
	Post-op infection	1
	Subsequent fracture/hardware failure	7
Type of nonunion	Atrophic	21
	Hypertrophic	4
	Oligotrophic	28
Septic nonunion	Yes	9
	No	44
Nonunion treatment	Operative	43
	Nonoperative	11
	Lost to follow up	1
Type of surgery	ORIF	40
	IMN	1
	External fixation	1
	Antibiotic spacer	1
	rTSA with long stem	1
Approach	Anterior	38
	Posterior	6

9 patients were found to have positive cultures at time of revision (Table 3). Negative intra-operative cultures at the time of revision was associated with as shorter time to healing of the nonunion after surgery (198 ± 177.54 vs 451.25 ± 314.377 days, *p* = 0.034). There were no significant differences in the number of revision procedures, time from injury to nonunion, or time from injury to revision surgery comparing those patients with and without positive cultures at time of revision.

**Table 3:** Intraoperative culture status, revision burden, and temporal parameters in humeral shaft nonunion

Group Statistics	Positive culture	N	Mean (days)	Std. Deviation	*p*-value
Time from injury to nonunion surgery	Yes	7	140.71	102.851	0.672
	No	34	163.59	133.625	
Time from injury to nonunion	Yes	9	119.78	96.321	0.634
	No	50	136.52	96.584	
Time from nonunion surgery to healed	Yes	4	451.25	314.377	0.034
	No	19	198.37	177.549	
Number of Revision Procedures	Yes	7	1.86	1.574	0.07
	No	21	0.9	0.995	

Patients were treated with some form of bone graft at time of revision (Table 4).

**Table 4:** Bone graft usage, revision burden and temporal parameters in humeral shaft nonunion

	Yes Bone graft			No Bone graft			*p*-value
	Valid N	Mean	SD	Valid N	Mean	SD	
Number of Revision Procedures	26	1.19	1.23	2	0.5	0.71	0.445
Time from injury to nonunion surgery	38	161.92	132.52	3	131.33	41.06	0.696
Time from injury to nonunion	41	126.05	94.52	18	152	99.32	0.343
Time from nonunion surgery to healed	21	255.43	227.3	2	105	25.46	0.37

42 patients (77.78%) required only one revision procedure before going on to radiographic healing of their nonunion (Table 5). 12 patients required more than one revision procedure. Patient demographics, initial injury characteristics, nonunion characteristics, use of bone graft, and time to nonunion diagnosis were not significant comparing between those that required 1 vs. more than 1 revision procedure. Patients with more than one revision procedure had longer follow-up (846.25 ± 624.75 days vs. 358 + 261.65 days, *p* < 0.001) and were slower to heal (433 ± 388.75 days vs. 202.21 ± 157.38 days, *p* = 0.028).

**Table 5:** Temporal outcomes separated by degree of revision burden in humeral shaft nonunion

	2 or more revisions			1 revision only			*P*-value
	N	Mean	SD	N	Mean	SD	
# of revision procedures	12	2.17	1.11	16	0.38	0.5	< 0.001
Duration of follow up (days)		846.25	624.75		358.05	261.65	< 0.001
Time from injury to nonunion surgery (days)		182.5	155.12		152.32	120.04	0.262
Time from injury to nonunion (days)		123.58	84.76		136.52	101.3	0.344
Time from nonunion surgery to healed (days)		433	388.75		202.21	157.38	0.028

## Discussion

In this single-center retrospective series, no statistically significant differences were detected in terms of healing time or number of reoperations amongst those patients treated with revision with or without bone grafting. However, positive cultures at initial revision, and requirements for more than one revision procedure were associated with delayed healing.

Multiple studies identify nonoperative initial fracture management as a risk factor for primary nonunion [1, 6, 12]. One study of 126 humeral shaft fractures additionally suggested that early operative fixation (as distinguished from late surgery which follows a period of nonoperative treatment) was associated with a higher union rate [6]. Conversely, Olson et al. observed similar rates of primary fracture union between nonoperative and operative groups, even when the union of only closed fractures treated with ORIF were analyzed [16]. Two studies used the Disabilities of Arm, Shoulder and Hand score as the primary outcome, finding that operative treatment of humeral shaft fractures compared to functional bracing did not significantly improve function [15, 17]. However, both studies secondarily demonstrate high rates of nonunion and/or secondary surgery in patients treated nonoperatively [15, 17]. Indeed, this cohort of nonunion was isolated from a larger series, in which initial nonoperative management, comorbid alcoholism or hypothyroidism, and specific surgical implant configurations were described as risk factors for humeral shaft nonunion [4].

Septic nonunion presents a distinct challenge to treatment. Septic nonunion can be difficult to identify as intraoperative cultures are not routinely collected at all institutions. Several studies in the literature intended to exclude septic nonunion, but either unexpectedly discovered positive cultures or retrospectively questioned patients’ initial aseptic designations due to high rates of postoperative infection [18–20]. These fracture-related infections are often subclinical, bearing non-specific radiographic signs and laboratory indicators such as C-reactive protein, erythrocyte sedimentation rate and white blood cell count [21–24]. Nine patients in this investigation with history of operative initial fixation were found to have positive cultures at time of revision for nonunion and experienced disproportionately prolonged healing compared to those without positive cultures. This finding aligns with a study by Haase et al. where 28 of 122 patients with long bone nonunion were septic, and the septic group time to union was an average of 129 days longer [25]. Olszewski et al. also observed a significant difference in healing rate between culture-positive and culture-negative patients where 20% of 666 long bone nonunions had positive cultures in the absence of positive inflammatory markers [20].

Many studies combine atrophic and oligotrophic categories for analysis, likely due to similar radiographic appearance and limited biologic activity. Atrophic/oligotrophic nonunion was the most common across multiple large retrospective studies, accounting for 70–85% of the humeral shaft nonunions analyzed [9, 11, 18, 19]. Indeed, 49/53 (92.5%) of the present series were atrophic or oligotrophic. Insufficient native biology is often considered an indication to utilize bone grafts; autogenous bone augmentation fulfills both criteria for biology and structural support, and therefore is frequently used in conjunction with compression plating for treatment of humeral shaft nonunion [5]. The iliac crest is a popular autograft source; though, due to rates of subsequent donor site morbidity (i.e., pain and infection) reaching 38–44%, usage of allografts such as demineralized bone matrix (DBM) with or without bone morphogenetic protein (BMP) are increasing with little difference in union rate or patient-reported outcomes [5, 10–12]. However, several studies have shown no association between nonunion type and union rate, further challenging the need for routine bone graft usage in humeral diaphyseal nonunion [9, 11]. In this cohort, the bone graft utilized were heterogeneous, and any meaningful comparisons limited by the size of the cohort described.

12 of the 54 patients treated for nonunion went on to require > 1 revision surgery. These patients could not be differentiated from patients whose nonunion healed after one revision procedure by the factors able to be considered in this analysis. Return to the operating room following failed nonunion repair often serves as a proxy for recalcitrant nonunion; a recent study by Roddy et al. further defined it as hardware failure and/or persistent lack of radiographic signs of healing a minimum of one year from surgery with pain. Using this definition and assessing the outcomes of 159 patients with humeral diaphysis nonunion, Roddy et al. similarly found no association between patient demographics, use or type of bone graft and recalcitrant nonunion [19]. However, they found an increased risk of recalcitrant nonunion with each successive surgery from the initial nonunion procedure; though, notably, this was not independently associated with initial operative treatment of the acute fracture. In another study of 125 patients, a stepwise correlation between risk of > 1 nonunion surgery and total number of procedures was highlighted [12]. Initial nonoperative treatment was identified as a risk factor for nonunion, and plate fixation of the initial fracture predicted > 1 surgical procedure for nonunion [12]. Additionally, Roddy et al. observed a correlation between atrophic or oligotrophic nonunion and > 1 revision surgery [19]. Further, in a cohort of 90 patients with aseptic humeral nonunion following initial operative fixation, with recalcitrant nonunion defined as reoperation for nonunion regardless of time, failure to meet criteria for successful union a minimum of 180 days following surgery or fixation failure, patients who did not undergo revision internal fixation and those with infection were more likely to develop recalcitrant nonunion [18].

Humeral shaft nonunion remains a complex clinical problem with evidence in support of many factors contributing to nonunion development and healing. The existing varying definitions of recalcitrant nonunion illustrate a lack of consensus in the current literature regarding optimal timing of diagnosis and forms of treatment for these nonunion, although risk factors for prolonged recovery have been described, including in the setting of oligotrophic/atrophic nonunion or infection. The present analysis find agreement, suggesting positive intraoperative cultures and requirements for > 1 revision procedure, indicating recalcitrant nonunion, as significant in risk of healing delay.

### Limitations

This study has several limitations. The variability of injuries and patient data available for retrospective review, including types of bone grafts and nonunion, patient chart detail and length of follow-up, limited the ability to examine this data by sub-group analysis and via more specific statistical methods. The generalizability of the study is also limited by sample side, lack of functional outcomes, and presentation of the data from one academically affiliated institution. Finally, regarding patients requiring more than one revision, conclusions were limited by small sample size and patient factors beyond the scope of this analysis requiring recruitment and follow-up ideally via future prospective analysis.

## Conclusion

Humeral shaft nonunion remains a complex clinical problem. In this analysis, positive cultures at time of initial revision, and requirements for > 1 revision procedure were found be significantly associated with delayed healing. Use of bone graft at time of revision was not significantly associated with faster healing or decreased likelihood of requiring > 1 revision procedure. No other specific patient or injury characteristics were significantly associated with an increased likelihood of requiring > 1 revision procedure.

## Data Availability

Data is available upon reasonable request to the corresponding author.
